# Short-Term Outcomes of Outpatient Endoscopic Resection for Selected Superficial Gastric Neoplasms: A Predominantly EMR-Based Real-World Cohort Study

**DOI:** 10.3390/jcm15197411

**Published:** 2026-09-24

**Authors:** Seung-Jun Kim, Chul-Hyun Lim

**Affiliations:** 1 Department of Gastroenterology, CHA Bundang Medical Center, CHA University School of Medicine, 59 Yatap-Ro, Bundang-Gu, Seongnam 13496, Republic of Korea; gmt475@gmail.com; 2 Division of Gastroenterology, Department of Internal Medicine, Eunpyeong St. Mary’s Hospital, College of Medicine, The Catholic University of Korea, 1021 Tongil-Ro, Eunpyeong-Gu, Seoul 03312, Republic of Korea

**Keywords:** stomach neoplasms, endoscopic mucosal resection, endoscopic submucosal dissection, ambulatory surgical procedures, adverse events

## Abstract

**Background/Objectives:** Although same-day discharge after gastric endoscopic resection has been explored, contemporary data on outpatient treatment using predominantly endoscopic mucosal resection (EMR)-based techniques remain limited. This study aimed to describe the technical and short-term clinical outcomes of outpatient endoscopic resection for selected superficial gastric neoplasms. **Methods:** We retrospectively reviewed 259 superficial gastric neoplasms from 254 consecutive patients who underwent outpatient endoscopic resection. The primary outcomes were short-term adverse events and unplanned healthcare utilization, whereas en bloc and R0 resection were evaluated as secondary technical outcomes. **Results:** The mean age was 69.1 ± 10.1 years. The cohort predominantly comprised low-grade dysplasia (81.1%) and small lesions (mean endoscopic size, 9.7 ± 3.9 mm). EMR-based techniques accounted for 94.2% of resections, whereas endoscopic submucosal dissection (ESD) accounted for 5.8%. Three patients (1.2%) experienced short-term adverse events: one developed postprocedural bleeding (0.4%), and two experienced severe abdominal pain (0.8%). All three patients visited the emergency department, and the two patients with abdominal pain required unplanned hospital admission; no perforation occurred. All patients completed the scheduled outpatient follow-up visit. En bloc and R0 resection rates were 96.1% and 95.0%, respectively. **Conclusions:** In this selected EMR-predominant cohort, short-term adverse events and unplanned hospital admissions were uncommon, and en bloc and R0 resection rates were high. Prospective comparative studies are warranted.

## 1. Introduction

According to current clinical practice guidelines, including those of the Japanese Gastroenterological Endoscopy Society (JGES), the American Society for Gastrointestinal Endoscopy (ASGE), and the European Society of Gastrointestinal Endoscopy (ESGE), endoscopic mucosal resection (EMR) and endoscopic submucosal dissection (ESD) are recommended as standard endoscopic treatment options for superficial gastric neoplasms [1,2,3]. These minimally invasive procedures enable effective local treatment while preserving gastric function.

However, postprocedural bleeding (PPB) remains the most common adverse event after gastric endoscopic resection, with a pooled incidence of approximately 5.1% reported after gastric ESD [4] and delayed bleeding reported in 5.3% of patients in a large series of gastric EMR [5]. Because PPB may result in clinically significant consequences, routine postprocedural observation in the hospital remains common in many clinical settings. Accordingly, recent clinical practice updates and predictive models have focused on stratifying bleeding risk to guide postprocedural management and determine the appropriate level of observation for individual patients [6,7].

Routine admission, however, increases healthcare resource utilization and costs. Advances in endoscopic techniques and prophylactic coagulation of visible vessels may reduce procedure-related complications [8,9]. Acid-suppressive therapy is also routinely used for the management of artificial ulcers after gastric endoscopic resection [10,11]; however, its contribution to clinical outcomes in outpatient management remains uncertain.

A recent Western multicenter prospective study evaluated same-day discharge after ESD in appropriately selected patients [12]. In Korea, Ahn et al. compared day-patient and inpatient gastric ESD for small antral neoplasms and reported comparable short-term outcomes [13]. However, that study focused on ESD, whereas contemporary real-world data on outpatient resection using predominantly EMR-based techniques across a broader range of gastric locations remain limited.

Therefore, this descriptive study aimed to characterize short-term adverse events, unplanned healthcare utilization, and technical outcomes after outpatient endoscopic resection in a selected cohort of superficial gastric neoplasms treated predominantly with EMR-based techniques.

## 2. Materials and Methods

### 2.1. Patients and Study Design

This retrospective, single-center study included 259 histopathologically confirmed superficial gastric neoplasms from 254 consecutive patients who underwent outpatient endoscopic resection at Eunpyeong St. Mary’s Hospital between June 2020 and December 2025. Short-term adverse events and unplanned healthcare utilization were analyzed on a per-patient basis, whereas technical outcomes were analyzed on a per-lesion basis. During the study period, 707 patients underwent gastric endoscopic resection at our institution, of whom 380 (53.7%) were managed on an outpatient basis and 327 (46.3%) on an inpatient basis. Because this study was designed to describe outpatient practice, inpatient procedures were not analyzed, and the exclusion criteria described below were applied only to the outpatient cohort. No formally documented institutional eligibility protocol was in place during the study period. Outpatient management was determined by the treating endoscopist before the procedure, based on consistently applied clinical considerations including estimated lesion size and anticipated technical difficulty, bleeding risk assessed using the BEST-J score, comorbidity burden and antithrombotic use, and social factors such as caregiver availability and the ability to return promptly to the hospital if delayed symptoms occurred. The BEST-J score was calculated for each patient using the originally reported variables and weighting, and patients were categorized according to the published risk strata; because only a small number of patients were classified as high or very high risk, these two categories were combined for reporting. Patients considered to be at low to intermediate bleeding risk were generally managed on an outpatient basis. A small number of high-risk patients also underwent outpatient treatment after individualized risk assessment, including specialist consultation regarding the temporary interruption of antithrombotic therapy. Lesions were excluded if they were non-neoplastic, had incomplete medical records, were identified in patients with a history of previous gastric surgery, required non-elective endoscopic resection, or were identified in patients with coagulopathy. The study protocol was approved by the Institutional Review Board of Eunpyeong St. Mary’s Hospital (IRB No. PC26RISI0018), and the requirement for informed consent was waived because of the retrospective study design. The study selection process is summarized in Figure 1.

Of the 707 patients who underwent gastric endoscopic resection during the study period, 380 (53.7%) were managed on an outpatient basis and constituted the study population; 327 (46.3%) were managed on an inpatient basis and were not analyzed. Among 408 lesions in the outpatient cohort, 149 were excluded, including non-neoplastic lesions (*n* = 127), incomplete medical records (*n* = 6), history of previous gastric surgery (*n* = 8), non-elective endoscopic resection (*n* = 7), and coagulopathy (*n* = 1). Finally, 259 superficial gastric neoplasms from 254 patients were included in the analysis.

### 2.2. Endoscopic Procedures

Endoscopic resection was performed using standard therapeutic endoscopic equipment by four endoscopists, each certified by the Korean Society of Gastrointestinal Endoscopy and with more than 10 years of experience in therapeutic endoscopy. The resection method was selected at the discretion of the endoscopists according to lesion size, morphology, and location. Procedures were categorized as conventional EMR (cEMR), precut EMR (EMR-P), ligation-assisted EMR (EMR-L), or endoscopic submucosal dissection (ESD). cEMR was defined as snare resection after submucosal injection without adjunctive devices. EMR-P was defined as snare resection after circumferential or partial precutting. EMR-L was defined as EMR performed with a ligation device. ESD was defined as a resection technique involving circumferential mucosal incision followed by submucosal dissection with a dedicated knife. At the completion of endoscopic resection, prophylactic coagulation of visible vessels in the artificial ulcer was performed routinely and, given the outpatient setting, was applied more liberally than is typical in inpatient practice: any exposed or protruding vessel identified at the ulcer base was coagulated regardless of its appearance. Because this step was an integral part of the resection technique and was not recorded as a discrete procedural item, the proportion of lesions in which it was applied could not be determined retrospectively.

### 2.3. Periprocedural Management

Conscious sedation with midazolam and pethidine was used in 250 patients (98.4%), whereas 4 patients (1.6%) underwent the procedure without sedation. After the procedure, all patients, including those undergoing ESD, underwent routine post-sedation recovery and were observed for approximately 1 h according to the same institutional protocol, based on the Korean Society of Gastrointestinal Endoscopy guidelines. Before discharge, abdominal radiography was performed to screen for procedure-related perforation. Discharge criteria included stable vital signs, adequate recovery from sedation, absence of clinically significant abdominal pain, bleeding, or peritoneal signs, and no radiographic evidence of perforation. Before discharge, patients received written instructions regarding diet, medication use, warning symptoms, and the need for immediate medical evaluation in the event of hematemesis, melena, persistent abdominal pain, dizziness, or fever. Urgent evaluation was available through the institutional emergency department and on-call medical service. All patients were scheduled for an outpatient follow-up visit 2–4 weeks after the procedure to review the histopathology results and assess delayed adverse events. Delayed complications were ascertained at this scheduled visit and supplemented by a review of emergency department visits and unplanned hospital admissions during the follow-up period.

Acid-suppressive therapy was administered periprocedurally using either a proton pump inhibitor (PPI) or a potassium-competitive acid blocker (P-CAB). The principal PPI and P-CAB regimens were lansoprazole 30 mg once daily and tegoprazan 50 mg once daily, respectively. When treatment was initiated before the procedure, PPI therapy was started 4 days before endoscopic resection, whereas P-CAB therapy was started 2 days before the procedure. In patients who were already receiving these medications, the pre-existing regimen was continued. Postprocedural therapy was generally maintained for at least 4 weeks and was extended up to 8 weeks in cases with a large artificial ulcer. Antithrombotic agents were managed according to the clinical practice guidelines of the Korean Society of Gastrointestinal Endoscopy for the periprocedural use of antithrombotic agents [14]. Antiplatelet agents were withheld for 5–7 days before the procedure. In patients receiving warfarin, the procedure was performed after confirming an international normalized ratio below 1.5, and direct oral anticoagulants were withheld for 2 days. In patients considered to be at high thromboembolic risk, the decision to interrupt therapy was individualized in consultation with the prescribing cardiologist or neurologist. Antithrombotic therapy was resumed after the procedure once no evidence of bleeding was observed.

### 2.4. Histopathologic Evaluation

Resected specimens were fixed in formalin and examined histopathologically by board-certified gastrointestinal pathologists. Histologic diagnosis, resection margin status, pathologic tumor size, and resection specimen size were recorded from the pathology reports.

### 2.5. Variables and Outcomes

Baseline variables included age, sex, body mass index, comorbidities, antithrombotic use, and periprocedural acid-suppressive therapy. Lesion-related variables included tumor location, endoscopic tumor size, resection specimen size, pathologic tumor size, gross morphology, histopathologic diagnosis, and resection method.

The primary clinical outcomes were short-term adverse events and unplanned healthcare utilization occurring between the procedure and the scheduled outpatient follow-up visit 2–4 weeks later. Unplanned healthcare utilization was defined as any procedure-related emergency department visit or unplanned hospital admission. Adverse events were prespecified and included postprocedural bleeding, perforation, and severe abdominal pain. Other events, such as sedation-related cardiopulmonary complications, fever, and aspiration, were not prespecified and were not systematically assessed. Severe abdominal pain was defined as postprocedural abdominal pain rated ≥7 on a 0–10 numeric rating scale (NRS). Postprocedural bleeding was defined, in accordance with criteria commonly used in previous studies of bleeding after gastric endoscopic resection [4,6], as clinically evident bleeding after the procedure, such as hematemesis or melena, accompanied by a decrease in hemoglobin level of >2 g/dL, the need for endoscopic hemostasis, or both. Secondary technical outcomes were en bloc resection and resection margin status. En bloc resection was defined as removal of the lesion in a single piece. R0 resection was defined as histologically negative lateral and vertical margins, R1 resection as histologic involvement of either margin, and Rx resection as an indeterminate margin status. Resection specimen size was defined as the maximal diameter documented in the pathology report.

### 2.6. Statistical Analysis

Continuous variables are presented as mean ± standard deviation, and categorical variables are presented as number (%). Given the small number of adverse events, analyses were descriptive, and no inferential comparisons among resection techniques or formal risk-factor analyses were performed. Exact binomial 95% confidence intervals were calculated for the primary clinical outcome rates. Statistical analyses were performed using IBM SPSS Statistics for Windows, version 29.0 (IBM Corp., Armonk, NY, USA).

## 3. Results

### 3.1. Baseline Clinical Characteristics

A total of 259 lesions from 254 patients were included in the analysis. The mean age of the patients was 69.1 ± 10.1 years, and 158 patients (62.2%) were male. The mean body mass index was 24.3 ± 3.1 kg/m^2^. Hypertension, diabetes mellitus, and dyslipidemia were present in 49.6%, 24.8%, and 39.0% of patients, respectively. According to the BEST-J score, 221 patients (87.0%) were classified as low risk, 28 (11.0%) as intermediate risk, and 5 (2.0%) as high or very high risk. Other baseline characteristics are summarized in Table 1.

### 3.2. Lesion Characteristics and Procedures

Most lesions were located in the antrum (64.1%), followed by the body (29.0%). The mean endoscopic and pathologic tumor sizes were 9.7 ± 3.9 mm and 8.0 ± 3.6 mm, respectively. The mean resection specimen size was 13.1 ± 5.2 mm, and 89.2% of specimens measured <20 mm. EMR-based techniques (cEMR, EMR-P, and EMR-L) accounted for 244 resections (94.2%), whereas ESD accounted for 15 (5.8%). The mean procedure duration was 7.1 ± 6.0 min and 6.1 ± 4.3 min for EMR-based techniques and 22.8 ± 8.0 min for ESD. Histologically, most lesions were low-grade dysplasia (81.1%), and adenocarcinoma was observed in 10.8% (Table 2).

### 3.3. Clinical and Technical Outcomes

Three patients (1.2%) experienced short-term adverse events. One patient developed postprocedural bleeding (0.4%) on the day after EMR-P and presented with melena. This patient had coronary artery disease, was receiving clopidogrel, and had received preprocedural PPI therapy. Clopidogrel had been withheld for 5 days before the procedure and was resumed on postprocedural day 1, on the same day that melena developed. Endoscopy showed no active bleeding but revealed a pigmented spot and a flat vessel at the ulcer base. Endoscopic hemostasis was performed for the visible vessel. Two patients experienced severe abdominal pain (0.8%) with NRS scores of ≥7 on postprocedural day 2. In both patients, abdominal computed tomography showed no evidence of perforation, and post-coagulation syndrome was diagnosed; both recovered with conservative management. All three patients visited the emergency department and were subsequently admitted; no perforation occurred. Detailed clinical information for the three patients with adverse events is provided in Table S1. All 254 patients completed the scheduled outpatient follow-up visit 2–4 weeks after the procedure. Beyond these events, no additional adverse events, emergency department visits, or unplanned hospital admissions were identified during the follow-up period (Table 3).

Regarding secondary technical outcomes, en bloc resection was achieved in 249 of 259 lesions (96.1%), and R0 resection was achieved in 246 of 259 lesions (95.0%). All 28 adenocarcinoma lesions were resected en bloc with R0 margins. Depth of invasion was T1a in 21 lesions (75.0%) and T1b in 7 (25.0%), and lymphatic invasion was identified in 1 lesion (3.6%); no lesion showed venous invasion or ulceration. According to the eCura system, 20 lesions (71.4%) were classified as eCura A and 2 (7.1%) as eCura B, whereas 6 (21.4%) were classified as eCura C-2. Additional gastrectomy was performed after three of these resections, and none showed residual tumor or lymph node metastasis in the surgical specimen; the remaining eCura C-2 lesions were managed with surveillance. Detailed histopathologic characteristics of the adenocarcinoma lesions are provided in Table S2. Among the 13 lesions with indeterminate margin status, 10 had been resected piecemeal, which precluded accurate assessment of the lateral margins, and the remaining 3 could not be evaluated because of cautery-induced artifact at the resection margin. All were non-adenocarcinoma lesions treated with EMR-based techniques, and none underwent repeat resection; all were managed with surveillance endoscopy at 6 months.

Outcomes according to resection technique are summarized in Table S3. Among the 244 lesions treated with EMR-based techniques, en bloc resection was achieved in 234 (95.9%) and R0 resection in 231 (94.7%), whereas all 15 ESD lesions were resected en bloc with negative margins. Two of the three adverse events occurred after EMR-based procedures and one after ESD. Because of the small number of ESD procedures and the small number of events overall, no statistical comparison was performed.

## 4. Discussion

In this retrospective, single-center cohort, short-term adverse events and unplanned healthcare utilization were uncommon after outpatient endoscopic resection for selected superficial gastric neoplasms. One postprocedural bleeding event occurred; no perforation was observed, and two patients required unplanned hospital admissions because of severe abdominal pain. Technical outcomes were also favorable, with en bloc and R0 resection rates of 96.1% and 95.0%, respectively. However, these findings should be interpreted in the context of a selected cohort consisting mainly of small lesions and low-grade dysplasia treated with EMR-based techniques. Therefore, the present study describes outcomes observed in a real-world outpatient practice setting and does not establish equivalence to inpatient management.

Previous studies of outpatient or same-day-discharge endoscopic resection have focused predominantly on ESD. A Western multicenter prospective cohort study reported the feasibility of same-day discharge after ESD in appropriately selected patients [12]. In Korea, Ahn et al. directly compared day-patient and inpatient gastric ESD for small antral neoplasms and reported comparable short-term outcomes [13]. In contrast, the present cohort was treated predominantly with EMR-based techniques, including cEMR, EMR-P, and EMR-L, whereas ESD accounted for only 5.8% of procedures. The study also included lesions arising across a broader range of gastric locations. Accordingly, the present findings complement previous outpatient ESD studies by providing contemporary real-world data from a predominantly EMR-based outpatient setting. Nevertheless, the absence of an inpatient comparator precludes conclusions regarding the comparative safety of outpatient and inpatient management.

The postprocedural bleeding rate in the present cohort was lower than the pooled incidence of approximately 5.1% reported after gastric ESD [4] and the 5.3% reported in a large series of gastric EMR [5]. These apparent differences, however, should not be interpreted as evidence that outpatient EMR-based treatment is safer than gastric ESD or inpatient care. The cited meta-analysis was derived from ESD cohorts treated frequently for larger lesions or early gastric cancers, whereas the present cohort predominantly comprised small lesions and low-grade dysplasia treated with EMR-based techniques. Reported bleeding rates after gastric EMR also vary widely, ranging from 0.38% in nationwide surveys to more than 10% in single-institution series, largely depending on how minor intraprocedural bleeding is classified [5]. Notably, a Korean study of EMR or ESD performed within a 2-day hospital stay reported delayed bleeding in 0.4% of 914 patients [15], a rate similar to ours, suggesting that short-stay management of selected patients may be associated with comparably low event rates. Furthermore, validated bleeding-risk models demonstrate that postprocedural bleeding is influenced by multiple patient- and procedure-related factors and that the underlying risk varies substantially among patients undergoing gastric endoscopic resection [6]. The low event rate observed in this study therefore likely reflects, at least in part, the selected characteristics and relatively low procedural complexity of the study population, which predominantly comprised low-grade dysplasia (81.1%) with a mean endoscopic lesion size of 9.7 mm; 98.0% of patients were classified as low or intermediate risk according to the BEST-J score. Because only three clinically relevant adverse events occurred, the event-rate estimates remain imprecise, and formal risk-factor analysis was not feasible. Notably, the single bleeding event occurred on the day clopidogrel was resumed, suggesting that the timing of antithrombotic resumption may warrant particular attention in the outpatient setting, where patients are not under direct observation during the early postprocedural period. A similar observation was reported in a Korean short-stay cohort, in which the single post-EMR bleeding event also occurred on the first day of antiplatelet resumption [15].

The high en bloc and R0 resection rates indicate that the selected lesions were technically amenable to the resection methods used in this cohort. All 28 adenocarcinoma lesions were also resected en bloc with histologically negative lateral and vertical margins. These findings suggest that selecting the resection technique according to lesion size, morphology, and location was associated with favorable technical outcomes. Nevertheless, en bloc and R0 resection primarily measure resection quality and do not directly determine whether outpatient management is appropriate. They should therefore be interpreted as secondary technical outcomes rather than as evidence supporting the safety of outpatient management or its equivalence to inpatient care. In addition, the small number of ESD procedures precludes meaningful conclusions regarding outpatient gastric ESD. Adenocarcinoma accounted for 28 lesions (10.8%) in this cohort, and outpatient resection of these lesions warrants explicit consideration. Resection technique was selected according to lesion characteristics rather than applied uniformly, and ESD was used in 7 of the 28 adenocarcinoma lesions (25.0%) compared with 5.8% of the overall cohort. These lesions were small (mean pathologic size 8.6 ± 4.5 mm) and predominantly antral, and 22 (78.6%) met eCura A or B criteria for curative resection. Importantly, curability is determined by histopathologic assessment after resection rather than at the time of the procedure; in the present cohort, all non-curative resections were identified at the scheduled follow-up visit and managed by additional gastrectomy or surveillance, and no surgical specimen showed residual tumor or lymph node metastasis. These observations suggest that outpatient management did not compromise or delay oncologic decision-making, although the small number of adenocarcinoma lesions precludes definitive conclusions.

The observed clinical outcomes should also be considered in the context of the periprocedural management used in this cohort, including selective prophylactic hemostasis and acid-suppressive therapy. Acid-suppressive therapy was initiated before the procedure, with different schedules for lansoprazole and tegoprazan reflecting institutional practice informed by the pharmacodynamic profiles of the two agents [16,17]. Previous studies have evaluated prophylactic coagulation of visible vessels as a strategy for reducing postprocedural bleeding after gastric endoscopic resection [9]. In the present cohort, prophylactic coagulation was applied more liberally than in conventional inpatient practice as a deliberate adaptation to the outpatient setting, and this may have contributed to the low bleeding rate observed. However, because its frequency was not recorded, this contribution could not be evaluated. P-CABs, including tegoprazan and vonoprazan, have also been evaluated for the treatment of artificial ulcers after gastric endoscopic resection, with favorable ulcer-related outcomes reported [10,11]. Most patients in the present cohort received a P-CAB; however, this study was not designed to compare P-CABs with PPIs, determine the optimal timing of treatment initiation, or assess the independent contribution of either regimen to clinical outcomes. Because P-CAB use was predominant and an appropriate comparator group was unavailable, the low adverse-event rate cannot be attributed to P-CAB therapy. Moreover, the size and endoscopic characteristics of the artificial ulcers were not directly assessed. The associations of acid-suppressive therapy, treatment-initiation timing, and artificial-ulcer characteristics with postprocedural outcomes should therefore be investigated in appropriately designed comparative studies.

This study has several limitations. First, it was a retrospective, single-center, uncontrolled descriptive study. Without an inpatient control group, the findings cannot establish whether outpatient management is equivalent or superior to inpatient management. Second, outpatient management was determined by the treating endoscopist rather than through a prespecified eligibility algorithm, and no formally documented institutional protocol was in place during the study period. This represents a substantial source of selection bias: patients with larger or technically demanding lesions, a higher comorbidity burden, or greater anticipated bleeding risk were preferentially managed as inpatients and are therefore absent from this cohort. The very low adverse-event rate observed here should accordingly be interpreted as reflecting this preferential selection at least as much as the outpatient strategy itself. Because inpatient procedures were outside the scope of this analysis, the magnitude and direction of this selection could not be quantified. Third, the study population consisted predominantly of small lesions and low-grade dysplasia treated with EMR-based techniques, and ESD accounted for only 15 lesions (5.8%). The stratified analysis shown in Table S2 is therefore descriptive only: a single adverse event in the ESD subgroup corresponds to 6.7%, illustrating that no meaningful estimate of outpatient ESD safety can be derived from 15 procedures. The findings should therefore not be generalized to larger neoplasms, technically complex procedures, or gastric ESD, which involves longer procedures, larger mucosal defects, and a substantially higher reported risk of delayed bleeding. Fourth, the small number of adverse events prevented inferential comparisons and formal identification of factors associated with bleeding or unplanned hospital admission. Fifth, although all patients completed the scheduled outpatient follow-up visit, the follow-up period was limited to 2–4 weeks after the procedure. Although emergency department visits and unplanned hospital admissions within the institution were reviewed, events managed exclusively at other institutions may not have been captured. Accordingly, long-term oncologic endpoints, including local recurrence, metachronous lesions, and survival, were not evaluated, as this study was designed to assess the short-term safety of the outpatient pathway rather than the oncologic outcomes of endoscopic resection itself. Finally, the absence of an appropriate PPI comparator and direct assessment of artificial-ulcer size prevented evaluation of the associations of acid-suppressive therapy and ulcer-related factors with postprocedural outcomes. Despite these limitations, the present study describes a consecutive contemporary cohort managed within an outpatient workflow and provides detailed information on lesion characteristics, resection techniques, technical outcomes, short-term adverse events, and unplanned hospital admissions. The findings may help define a clinical population suitable for further prospective evaluation. Future studies should use standardized outpatient eligibility and discharge criteria, include an appropriate inpatient comparator, and assess prespecified outcomes at both the patient and procedure levels. Multicenter studies will likely be required to obtain sufficient numbers of postprocedural bleeding events, emergency department visits, and unplanned hospital admissions for adjusted analyses. In addition to clinical outcomes, future studies should evaluate healthcare utilization, costs, patient satisfaction, and lesion-, procedure-, and ulcer-related factors.

## 5. Conclusions

Short-term adverse events and unplanned hospital admissions were uncommon in this selected, EMR-predominant cohort, and en bloc and R0 resection rates were high. However, these descriptive findings do not establish equivalence to inpatient management and should not be generalized to outpatient gastric ESD. Prospective comparative studies using standardized selection and discharge criteria are warranted.

## Figures and Tables

**Figure 1:**
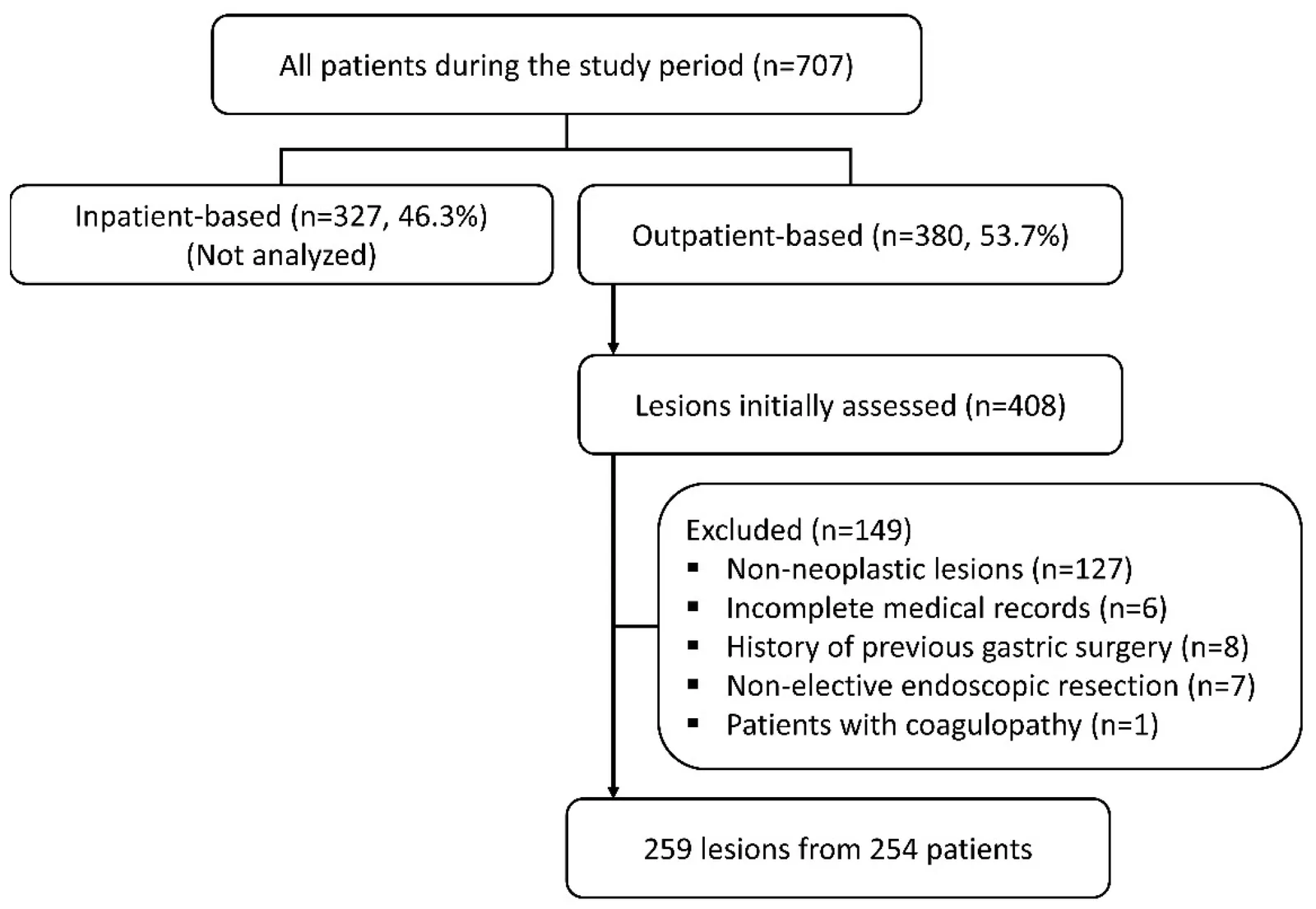
Study flow diagram of lesion selection.

**Table 1:** Baseline clinical characteristics of the study patients.

Characteristics	N = 254
Age (years), mean (±SD)	69.1 ± 10.1
Sex, *n* (%)	
Male	158 (62.2%)
Female	96 (37.8%)
Body mass index (kg/m^2^), mean (±SD)	24.3 ± 3.1
Comorbidities, *n* (%)	
Hypertension	126 (49.6%)
Diabetes mellitus	63 (24.8%)
Dyslipidemia	99 (39.0%)
Respiratory disease	6 (2.4%)
Chronic kidney disease	4 (1.6%)
Antithrombotic use, *n* (%)	
Antiplatelet agents	
Aspirin	20 (7.9%)
Clopidogrel	21 (8.3%)
Sarpogrelate	4 (1.6%)
Cilostazol	4 (1.6%)
Anticoagulants	
Warfarin	2 (0.8%)
Edoxaban	2 (0.8%)
Acid-suppressive regimen, *n* (%)	
PPI	34 (13.4%)
P-CAB	220 (86.6%)
BEST-J category, *n* (%)	
Low (0–1)	221 (87.0%)
Intermediate (2)	28 (11.0%)
High or very high (≥3)	5 (2.0%)

Abbreviations: PPI, proton pump inhibitor; P-CAB, potassium-competitive acid blocker.

**Table 2:** Lesion characteristics and endoscopic procedures.

Variables	N = 259
Tumor location, *n* (%)	
Cardia	3 (1.2%)
Body	75 (29.0%)
Angle	15 (5.8%)
Antrum	166 (64.1%)
Endoscopic tumor size (mm), mean (±SD)	9.7 ± 3.9
<10 mm (*n* = 128, 49.4%)	6.7 ± 1.4
≥10 mm (*n* = 131, 50.6%)	12.6 ± 3.3
Resection specimen size (mm), mean (±SD)	13.1 ± 5.2
<10 mm (*n* = 55, 21.2%)	7.4 ± 1.5
10–<20 mm (*n* = 176, 67.9%)	13.1 ± 2.5
20–<30 mm (*n* = 24, 9.3%)	22.5 ± 2.3
≥30 mm (*n* = 4, 1.5%)	34.3 ± 2.5
Pathologic tumor size (mm), mean (±SD)	8.0 ± 3.6
<10 mm (*n* = 169, 65.3%)	6.0 ± 1.8
≥10 mm (*n* = 90, 34.7%)	11.9 ± 2.8
Macroscopic morphology, *n* (%)	
Flat (IIb)	113 (43.6%)
Elevated (IIa, Is, Isp, IIa+IIb, SEL)	84 (32.4%)
Depressed (IIc, IIa+IIc, IIb+IIc)	62 (23.9%)
Resection method, *n* (%)	
Conventional EMR (cEMR)	60 (23.2%)
Precut EMR (EMR-P)	157 (60.6%)
Ligation-assisted EMR (EMR-L)	27 (10.4%)
Endoscopic submucosal dissection (ESD)	15 (5.8%)
Pathologic diagnosis, *n* (%)	
Low-grade dysplasia	210 (81.1%)
High-grade dysplasia	19 (7.3%)
Adenocarcinoma	28 (10.8%)
Well differentiated	21 (8.1%)
Moderately differentiated	6 (2.3%)
Poorly differentiated	1 (0.4%)
Neuroendocrine tumor	2 (0.8%)

SEL, subepithelial lesion. Two neuroendocrine tumors (0.8%) were included because they were neoplastic lesions managed within the same outpatient resection workflow, although they fall outside the strict definition of superficial gastric epithelial neoplasms.

**Table 3:** Short-term clinical and technical outcomes of outpatient endoscopic resection.

**Primary Clinical Outcomes, Patient-Level**	**N = 254**	**Exact 95% CI (%)**
Any prespecified adverse event	3 (1.2%)	0.24–3.41
Postprocedural bleeding	1 (0.4%)	0.01–2.17
Severe abdominal pain †	2 (0.8%)	0.10–2.82
Perforation	0 (0.0%)	0.00–1.44
Unplanned healthcare utilization		
Emergency department visits	3 (1.2%)	0.24–3.41
Unplanned hospital admissions	3 (1.2%)	0.24–3.41
**Secondary technical outcomes, lesion-level**	**N = 259**	
Resection pattern, *n* (%)		
En bloc	249 (96.1%)	—
Piecemeal	10 (3.9%)	—
Resection margin status, *n* (%)		
Complete (R0)	246 (95.0%)	—
Incomplete (R1)	0 (0.0%)	—
Indeterminate (Rx)	13 (5.0%)	—

† Severe abdominal pain was defined as an NRS score of ≥7. Abbreviations: CI, confidence interval. Primary clinical outcome categories were not mutually exclusive. All three patients who experienced an adverse event visited the emergency department and were subsequently admitted; the numbers of adverse events, emergency department visits, and unplanned hospital admissions therefore refer to the same three patients. An em dash indicates that the 95% CI was not calculated.

## Data Availability

The data presented in this study are available from the corresponding author upon reasonable request. The data are not publicly available due to patient privacy and institutional and ethical restrictions.

## Supplementary Materials

The following supporting information can be downloaded at: https://www.mdpi.com/article/10.3390/jcm15197411/s1, Table S1. Clinical course of the three patients with prespecified adverse events; Table S2. Histopathologic characteristics and eCura classification of the 28 adenocarcinoma lesions; Table S3. Outcomes according to resection technique.
