# Supplementary material for: Short-Term Outcomes of Outpatient Endoscopic Resection for Selected Superficial Gastric Neoplasms: A Predominantly EMR-Based Real-World Cohort Study
Source: J Clin Med. 2026 Sep 24;15(19):7411. doi: 10.3390/jcm15197411 (PMC13666126; doi:10.3390/jcm15197411)
Supplement: Supplementary file 1 [file jcm-15-07411-s001.zip › jcm-4526549-supplementary.pdf]

Table S1. Clinical course of the three patients with prespecified adverse events.

	**Patient 1**	**Patient 2**	**Patient 3**
**Adverse event**	Postprocedural bleeding	Severe abdominal pain	Severe abdominal pain
**Resection technique**	EMR-P	ESD	cEMR
**Location / Size (mm)**	Antrum / 7mm	Antrum / 20mm	Antrum / 12mm
**Onset**	Day 1	Day 2	Day 2
**Presenting symptom**	melena	Abdominal pain (NRS ≥7)	Abdominal pain (NRS ≥7)
**Hemoglobin change before / after (g/dL)**	12.2 / 9.8	—	—
**Diagnostic evaluation**	Emergency endoscopy	Abdominal CT	Abdominal CT
**Diagnosis**	Postprocedural bleeding	Post-coagulation syndrome	Post-coagulation syndrome
**Treatment**	Endoscopic hemostasis	Conservative treatment	Conservative treatment
**Transfusion**	No	—	—
**Hospital stay (day)**	4	5	6
**Clinical outcome**	Recovered	Recovered	Recovered

Abbreviations: CT, computed tomography; EMR-P, precut endoscopic mucosal resection; cEMR, conventional endoscopic mucosal resection; ESD, endoscopic submucosal dissection; NRS, numeric rating scale.

Table S2. Histopathologic characteristics and eCura classification of the 28 adenocarcinoma lesions.

**Variable**	**n (%)**
**Histologic type**	
Well differentiated	21 (75.0)
Moderately differentiated	6 (21.4)
Poorly differentiated	1 (3.6)
**Depth of invasion**	
T1a	21 (75.0)
T1b, <500 μm	3 (10.7)
T1b, ≥500 μm	4 (14.3)
**Lymphovascular invasion**	
Lymphatic invasion	1 (3.6)

Venous invasion	0 (0)
**Ulceration (UL1)**	0 (0)
**Resection outcome**	
En bloc resection	28 (100)
R0 resection	28 (100)
**eCura classification**	
A	20 (71.4)
B	2 (7.2)
C-2†	6 (27.4)
**Additional gastrectomy for eCura C-2 lesions**	3 (10.7)

†Of the six eCura C-2 lesions, three underwent additional gastrectomy; two patients declined surgery and one was managed with surveillance because of very advanced age. Abbreviations: eCura, endoscopic curability.

Table S3. Outcomes according to resection technique.

	EMR-based	ESD	Overall
**Lesion-level outcomes**	n = 244	n = 15	n = 259
En bloc resection, n (%)	234 (95.9)	15 (100)	249 (96.1)
Piecemeal resection, n (%)	10 (4.1)	0 (0)	10 (3.9)
R0 resection, n (%)	231 (94.7)	15 (100)	246 (95.0)
Indeterminate margin (Rx), n (%)	13 (5.3)	0 (0)	13 (5.0)
**Patient-level outcomes**	n = 239	n = 15	n = 254
Postprocedural bleeding, n (%)	1 (0.4)	0 (0)	1 (0.4)
Perforation, n (%)	0 (0)	0 (0)	0 (0)
Severe abdominal pain, n (%)	1 (0.4)	1 (6.7)	2 (0.8)
Any prespecified adverse event, n (%)	2 (0.8)	1 (6.7)	3 (1.2)
Unplanned hospital admission, n (%)	2 (0.8)	1 (6.7)	3 (1.2)
Emergency department visit, n (%)	2 (0.8)	1 (6.7)	3 (1.2)

Abbreviations: EMR, endoscopic mucosal resection; ESD, endoscopic submucosal dissection.
Footnote: EMR-based techniques comprised conventional EMR, precut EMR, and ligation-assisted EMR. No statistical comparison was performed because of the small number of ESD procedures and the small number of adverse events. Percentages in the ESD group should be interpreted with caution because of the very small denominator (n = 15), in which a single event corresponds to 6.7%.
